# The Actual Exactness of a Fast RMS Correction during Abrupt Voltage Change

**DOI:** 10.3390/s23042117

**Published:** 2023-02-13

**Authors:** Zekharya Danin, Ido Amiel, Neda Miteva, Moshe Averbukh

**Affiliations:** Department of Electrical/Electronic Engineering, Ariel University, Arial 40700, Israel

**Keywords:** AC voltage, RMS measurement, exactness, fault events

## Abstract

The requirement of RMS (voltage and current) measurements under a fraction of the AC period has become increasingly attractive in power systems. Some of these power applications are responsible for voltage stabilization in distribution lines when the voltage correction should be made in a short time, no more than one or two periods of the AC signal. Previously developed RMS correction applications must be validated in real-world situations characterized by an abrupt change (discontinuity) in voltage magnitude occurring even during a single AC period. Such circumstances can substantially influence the RMS estimation and, therefore, should be considered. This article suggests a mathematically based approach, validated in the laboratory, that improves the accuracy of a voltage RMS estimation for the appropriate measurement devices. It produces better results in cases where the RMS assessment should be done in a fraction of the AC period.

## 1. Introduction

The presence of private generating facilities, such as PV solar stations and individual generators, causes voltage regulation issues in distribution lines. The influence of fast-changing loads such as those widespread today in the electric grid from electrical vehicles, on the one hand, and stochastically generating plants (PV and wind power stations), together with faults occurring from time to time, produce a significant imbalance between consuming and generating power flows. Together with slow-reacting controllers (tap-changers) of transformers, such circumstances cause poor voltage stabilization, forcing grid managers to temporarily disengage some of the private stations. These situations lead to economic losses and should be prevented.

Numerous examples of research efforts have been made, and several technical solutions have been suggested. Among the suggested solutions, some are based on the usage of electrical storage [1,2,3,4], and some of them apply power flow dispatch at generating facilities [5,6]. Other solutions are based on the utilization of reactive power equipment [7,8,9,10,11,12,13,14]. Considering the influence of the high costs of high-voltage storage, reactive power usage as the voltage regulation model may be preferable among other methods. We have described the application of capacitive reactive power in a previous article [15]. A bank of capacitors connected in a special manner to a load during voltage drops can ensure the correction of voltage deviations during a half-hour AC period. However, the voltage adjustment to the standard level requires fast RMS estimation in a time frame of not more than a quarter of each AC period. In a short period of time, multiple methods for RMS assessments have been reported [16,17,18,19,20,21,22,23,24,25,26].

Muciek A. [16] submitted a method of RMS assessment based on the reconstruction technique of the rectified AC voltage average signals. The author claims an increase in the accuracy of the RMS measurement. Nonetheless, the method in principle employs modernized Fourier analysis, which necessitates a significant amount of computational power and is not always useful. Eduardo G. and Ribeiro E.R. [17] suggested a method for RMS estimation in situations characterized by slightly deviating AC frequency. Conventional techniques provide poor accuracy in these cases, and the proposed technique improves the RMS estimation. However, the method is based on the usage of digital signal processors (DSPs), which, as in the case of Fourier analysis, requires significant calculation power. Kyriazis G.A. [18] suggested RMS measurements with the application of a frequency-domain approach, which, as in [16], uses a Fourier expansion that is not effective in situations that can rely on relatively simple techniques. Shamin R. et al. [19] propose a robust technique for estimating voltage frequency and amplitude. The technique relies on a quadrature signal generator (QSG) to produce a fixed frequency. The generator applies a second order generalized integrator (SOGI) and an infinite-impulse-response differentiation filter (DF). The estimation technique is robust and computationally efficient. 

Raj N. and Pillay P. [20] proposed a relatively simple method for sag and swell estimation over a short period of time. However, this method needs to be implemented separately for detecting real RMS magnitude and, therefore, has restricted implementation. R. Cisneros-Magaña et al. [20] proposed a method that relies on time-domain methodology using the unscented Kalman filter. The method is used for the estimation of voltage sag magnitudes and time durations. F. Xiao et al. [22] describe a fast voltage detection method for low-voltage distribution systems having grid-tied renewable energy generation power plants. The voltage assessment approach consists of two stages. The first step includes signal prefiltering, after which the actual voltage detection is activated. Together with the virtual accuracy, the method suffers from the need to apply a two-stage voltage estimation process, which substantially increases the calculation times. A distinctive feature of the proposed technique is the use of a novelty designed orthogonal signal generator for the calculation of voltage amplitude and the RMS value. J-Maria F.-Arias et al. [23] suggested an RMS estimating technique based on a Z-domain moving window root mean square (RMS) evaluation function. As a result, the RMS voltage estimator avoids the inherent uncertainty of complex arithmetic operations related to the usual discretized RMS algorithm.

Barry G. Quinn [24] proposed a frequency and amplitude estimator using only three Fourier coefficients. An improved technique having an asymptotic variance of greater order is described. As a result, a new estimator is achieved, having an asymptotic variance more than 1.65 times faster than previous models.

P. and A. K. Singh [25] suggested a hybrid method to quantify the magnitude of the voltage sag. The results of the comparisons show that the proposed hybrid concept reduces the overestimation of sag magnitudes and duration, providing more precise detection of overshoot events. Amiel I. et al. [26] compared four different methods of the RMS assessment and suggested a novel one that relies on the RMS formulation with a correction coefficient. 

The present work analyzes the method of RMS estimation [26] during an abrupt voltage change and suggests a way for significant improvement to ensure the minimization of potential errors. The novelty of this work is in the broadening and significant improvement of the method’s application [26], when fast RMS assessment is required in events of sudden voltage change in high- and low-voltage distribution lines. The change is based on an improved mathematical representation of voltage changes in events. A simulation approach and real voltage events in distribution lines were used to validate this method. 

The article includes the following sections: (1) Introduction; (2) Method of fast RMS estimation based on a standard formulation with a correcting coefficient; (3) Results; (4) Discussion; and (5) Conclusions. 

Section 2 represents the analysis of the true accuracy of the fast RMS estimation suggested previously; Section 2.1 indicates typical patterns of abrupt voltage surges occurring in high-voltage distribution lines; Section 2.2 represents modeling of typical voltage distortions; Section 2.3 represents simulation of abrupt voltage alterations in PSIM [27] software; and Section 2.4 represents an improved mathematical approach for RMS estimation. Section 3 shows the results of the accuracy of the RMS assessment method. Section 4 summarizes the authenticity of the study and its results. Section 5 presents the conclusions of the article. 

## 2. Method

### 2.1. Typical Patterns of Abrupt Voltage Alterations in Distribution Lines

**Figure 1:**
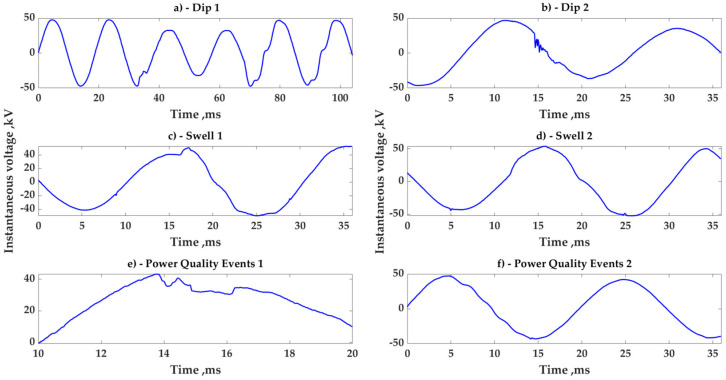
Different examples of voltage discontinuities in the distribution line: (**a**)—sudden voltage change, (**b**)—small flicks on voltage curve, (**c**,**d**)—abrupt voltage swell, (**e**)—voltage corruption, (**f**)—harmonics distortion.As can be seen, real voltage distortions are characterized by abrupt voltage alterations. The above types of real voltage distortions (Figure 1) were considered further for the modeling and mathematical developments of an improved measuring algorithm. All types of voltage discontinuities were analyzed for a common representation and verification of the submitted measurement technology. The appropriate modeling of such voltage distortion will be elaborated on in the next section.

Different types of events that cause sudden voltage changes make it difficult to precisely determine the actual RMS voltage amplitude during one or even one half of the AC period. The method presented in [26] is based on a mathematical description of a strict voltage sinusoidal curve and a correction coefficient obtained as per the requirement to assess RMS only in the periodic portion of the AC signal. However, real voltage curves show frequent non-ideality, sometimes distorting significantly from pure sine waves. These situations are generally caused by sudden load surges or fault events. Illustrations of such events are shown in the figures below. The authors recorded several examples of voltage non-ideality through the study of a specific high-voltage distribution line during the years 2019–2021. The summarized frequency of such events lies between 7 and 10% of the time, and this circumstance demonstrates the need for the enhancement of a voltage RMS estimation as submitted in [26]. Typical examples of voltage distortions are presented in Figure 1a–f.

### 2.2. Modeling of Abrupt Voltage Distortion

**Figure 2:**
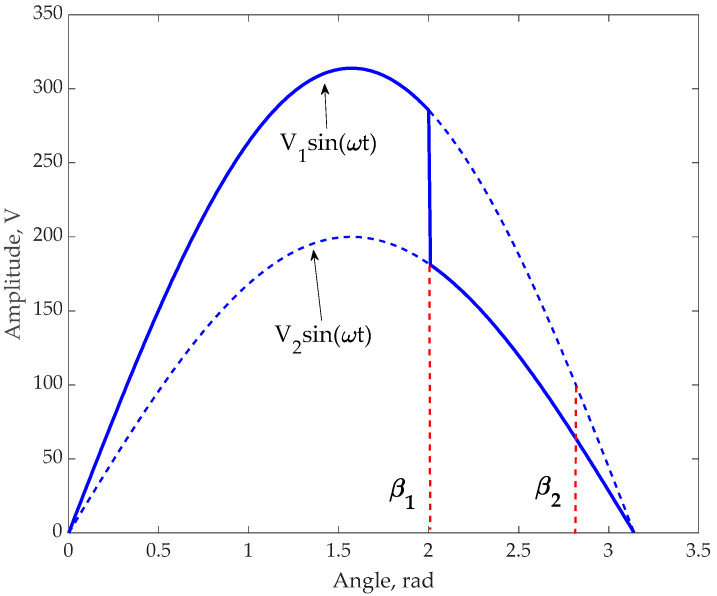
The model of voltage distortion occurring between the beginning and the end of a half-AC period.

The voltage distortion is modeled in this work as the inclusion of two voltages with the same frequency but with dissimilar amplitudes and RMS values and acting in series. The switch between these voltages occurs in time *t*_1_ from the beginning of some AC period. This time can be expressed as an angle *β*_1_. As per the requirement to improve the voltage value in the next AC half-period, the first step is RMS magnitude estimation from time *t*_1_ to *t*_2,_ which corresponds to two angles *β*_1_ and *β*_2_ (see Figure 2). Since the measurements of an instantaneous voltage magnitude should be finished before the end of the current half-AC period (angle equal to π), the last point of voltage estimation is designated as *β*_2_ < π. The time from the angle *β*_2_ (*t*_2_) to the end of a half-period (*t*_2_) is required to decide whether to correct the voltage or not. Such a representation of a voltage distortion reflects the real situation of voltage deterioration and will be used to output RMS expressions. 

To summarize the above discussion, the modeling of abrupt voltage distortion is provided by two sinusoidal signals having different amplitudes, where the first of them is changed by the second one inside the same half-AC period after implementation of a special phase angle. In this case, the RMS measurements should be ensured by mathematical manipulation of voltage magnitudes within the time from the beginning until reaching a phase angle that is less than the half-period of the AC signal.

### 2.3. Valid Technique of the RMS Estimation after Fast Voltage Alteration 

Mathematical expressions providing the RMS estimation of this model are represented below. As per the definition, the RMS value of a voltage between two points of time, *t*_1_ and *t*_2_, is:

(1) $$V_{rms}^{*}=\sqrt{\frac{1}{\omega \frac{\left(\beta_{2}-\beta_{1}\right)}{\omega }}\int_{t_{1}}^{t_{2}}V_{2m}^{2}sin^{2}\left(\omega t\right)d\left(\omega t\right)}\;$$

where *t*_1_ and *t*_2_ are the times corresponding to phase angles *β*_1_ and *β*_2_ in accordance with the relation: β=ωt.

It should be emphasized that the RMS value obtained in (1) is not exactly equal to the RMS value of the AC signal having amplitude *V*_1*m*_. The voltage magnitude correction (if needed) requires examination of the RMS from (1) vs. the RMS of a sinusoidal curve with amplitude *V*_1*m*_and the generation of a correcting coefficient for equalizing them. To relate two RMS magnitudes, the expression (1) should be modified. The transformation of (1) generates the expression.

(2) $$V_{rms}^{*}=\frac{V_{2m}}{\sqrt{2}}\sqrt{1-\frac{sin\left(\beta_{2}-\beta_{1}\right)cos\left(\beta_{2}+\beta_{1}\right)}{\left(\beta_{2}-\beta_{1}\right)}}=V_{2rms}\sqrt{1-\frac{sin\left(\beta_{2}-\beta_{1}\right)cos\left(\beta_{2}+\beta_{1}\right)}{\left(\beta_{2}-\beta_{1}\right)}}\;$$

The analysis of (2) provides the magnitude of the correcting coefficient *K_C_*_,_ relating one RMS value to another

(3) $$V_{1rms}=V_{rms}^{*}\cdot K_{C};\;\;\;\;\;\;\;\;\;\;K_{C}=\frac{1}{\sqrt{1-\frac{sin\left(\beta_{2}-\beta_{1}\right)cos\left(\beta_{2}+\beta_{1}\right)}{\left(\beta_{2}-\beta_{1}\right)}}}\;$$

**Figure 3:**
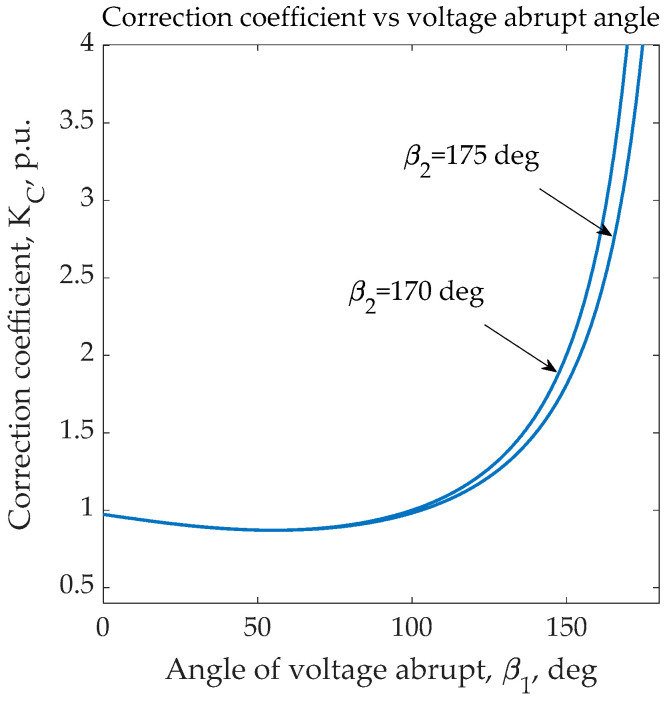
The correction coefficient *K_C_* vs. an angle of a voltage discontinuity for two values of *β***_2_** equal to 170° and 175°.

The value of $V_{rms}^{*}$ can be obtained numerically by summing the squares of the instantaneous voltage values measured with a specific time resolution from *t*_1_ to *t*_2_. This procedure is the simplest and is sufficiently accurate for use in an RMS control scheme. Below (Figure 3) are shown graphs plotting *K_C_* vs. angle *β*_1_ for two values of angle *β*_2_ equal to 170° and 175°.

The calculation of the RMS voltage after a fast change should be accomplished by the technique for the RMS estimation performed during the entire AC half-period, including the distorted sine curve. In fact, the closeness of angle *β*_1_ to a maximum admissible value of *β*_2_ increases the correction coefficient *K_C_* and decreases the number of instantaneous voltage points for the RMS estimation. Given the statistical errors of both factors, errors in RMS estimation may be significantly magnified, resulting in the loss of stability of a control system responsible for voltage consistency. Therefore, if a fast voltage change occurs close to the end of the AC half-period, it should change the RMS estimation. This circumstance necessitates an evaluation over the entire half-period rather than just the points measured after the voltage discontinuity.

### 2.4. The RMS Estimation When Fast Voltage Alterations Are Present in a Half-Period of an AC Signal

The formal RMS definition when two different sinusoidal signals are changing relative to one another inside an AC signal after angle α can be presented as follows:

(4) $$V_{rms}=\sqrt{\frac{2}{\omega T}\int_{0}^{\frac{T}{2}}V^{2}\left(\omega t\right)d\omega t=\frac{2}{\omega T}\left[V_{1m}^{2}\int_{0}^{\alpha }\sin^{2}\left(\omega t\right)d\omega t+V_{2m}^{2}\int_{\alpha }^{\pi }\sin^{2}\left(\omega t\right)d\omega t\right]}\;$$

After transformation

(5) $$V_{rms}=V_{1rms}\sqrt{\left(\frac{\alpha }{\pi }+K_{21}\left(1-\frac{\alpha }{\pi }\right)\right)+\frac{\sin2\alpha }{2\pi }\left(1-K_{21}\right)}\;$$

where $K_{21}={\left(\frac{V_{2rms}}{V_{1rms}}\right)}^{2}$.

**Figure 4:**
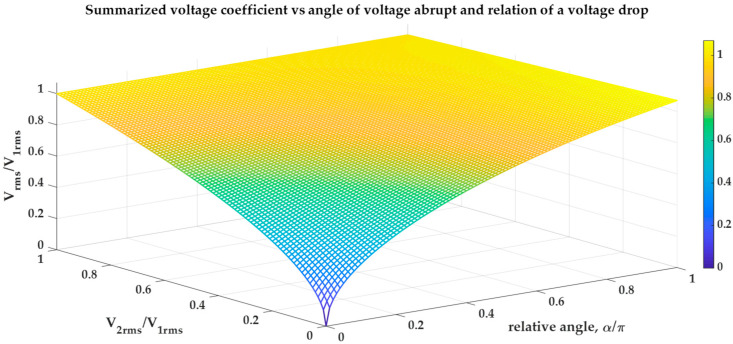
Summarized voltage coefficient vs. angle of a voltage discontinuity and voltage relation.

The graph of the relative voltage RMS because of the angle due to the voltage discontinuity is presented in Figure 4.

In Figure 4, we can see that the closer the switching angle α is to zero, the more influence voltage *V*_2_ has on the determination of the entire RMS value. However, this is not a linear relationship, and the influence of voltage *V*_2_ grows faster at the beginning of a switching angle than when it is close to the angle π.

### 2.5. Analysis of the Accuracy for the RMS Assessment Method

The accuracy of the RMS assessment plays a significant role in a control system responsible for AC voltage stability. A significant error in the fast estimation of RMS during a half-period can diminish the stability of a control system and lead to an inappropriate oscillation of correcting control signals and a total loss of functionality. The accuracy of the RMS assessment for the complex signal including a minimum of two sinusoids with different amplitudes (see Figure 2) strongly depends on the range between angles *β*_1_ and *β*_2_. The closer *β*_1_ is to *β*_2_ the less the number of measured instantaneous voltage points. Additionally, the assessment of a signal’s RMS after a voltage discontinuity and, therefore, in the period following this event is dependent on the correction coefficient *K_C_*. The RMS is calculated based on the summed squared magnitudes multiplied by *K_C_*_,_ whose value is increasing when *β*_1_ is close to *β*_2_ achieving values of 4–6 and above. Thus, taking into consideration the stochastic nature of voltage measurement errors, the dependence of the RMS deviation from its exact value should be estimated. In principle, a significant error in the RMS assessment can lead to the application of the procedure in Section 2.4 instead of that suggested in Section 2.3.

The accuracy is investigated under the assumption that voltage measurement errors are distributed according to the gamma function. The choice of gamma distribution law was determined for several reasons. First, it is always a positive function, and only positive magnitudes can be obtained in the voltage measurements. Second, it is close to the Gaussian function and is responsible for the description of most stochastic processes, including measurements of electrical parameters. Besides, the gamma function is closer to the Gaussian curve as the dispersion of a group statistic becomes smaller. This situation is usually correct and typical for measurements performed by modern devices. The gamma distribution function [27] of any variable x is defined by two parameters, the *k*-shape parameter (*k* > 0), and *θ*-scale parameter (*θ* > 0), and is represented as follows:

(6) $$f\left(x\right)=\frac{1}{\Gamma \left(k\right)\theta^{k}}x^{k-1}e^{-\frac{x}{\theta }},$$

where Γ*(x)* is a gamma-function: $\Gamma \left(k\right)=\int_{0}^{\infty }t^{k-1}e^{-t}dt$.

The average value of variable x is determined by *x_av_* = *kθ*, and variance *σ_x_ = kθ^2^.*

Define the time resolution of one instantaneous voltage measurement as Δ*t*. The typical Δ*t*-value achievable for high voltage is approximately ~10 μs. The total number of points for the 50 Hz signal in the half-period is equal to $N_{max}=\frac{T}{2\Delta t}=1000.\;$As angle *β*_1_ (the angle of sudden voltage change) increases, the total number of points for RMS estimation decreases. Define *σ_v_* as the standard deviation (STD) in each measurement. The STD in an assembly of N-points is

(7) $$\sigma_{N}=\frac{\sigma_{v}}{\sqrt{N}}$$

However, this STD should be multiplied by the correction coefficient since real voltage assessment is done using the same multiplication. This correction coefficient can be calculated as per (3), and it grows as *β*_1_ approaches angle *β*_2_. Moreover, during the approach from *β*_1_ to *β*_2,_ the summed STD value increases as N decreases. Ultimately, this causes an increase in measurement error and should be avoided. If this error is too great, the RMS assessment should be done according to (2.4).

As an example, the situation with *σ_v_ =* 10%, Δ*t =* 10 μs, and *β*_2_ *=* 175° was analyzed, and the graph of a relative STD error is shown below. It is worth noting that the angle *β*_2_ *=* 175° signifies the 0.5 ms time for the solution if a correction of the RMS of a voltage is done.

**Figure 5:**
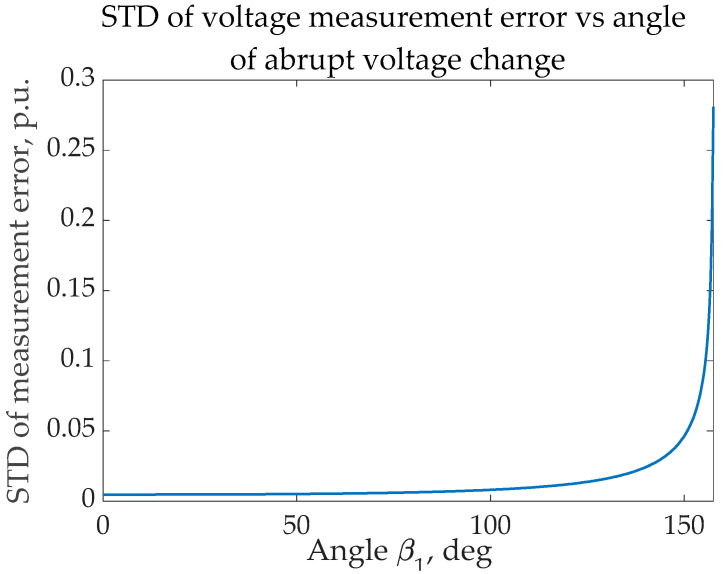
The example of the error STD during voltage RMS measurements.

A brief analysis of the graph Figure 5 shows evidence of low accuracy after angle *β*_1_ crosses 150°. Therefore, the acquisition of voltage magnitudes for the RMS estimation when the typical STD of measurement errors is close to 10% can be carried out only until 150°.

### 2.6. Simulation of Abrupt Voltage Alterations in PSIM

**Figure 6:**
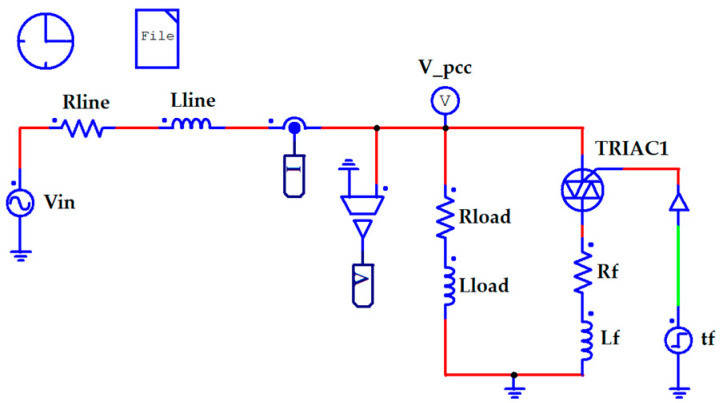
The scheme of the PSIM model for simulating voltage discontinuity.

The improvements to a method for RMS assessment should be initially verified in simulation. This is done by PSIM [28] software, and the appropriate simulating circuit is shown in Figure 6.

**Figure 7:**
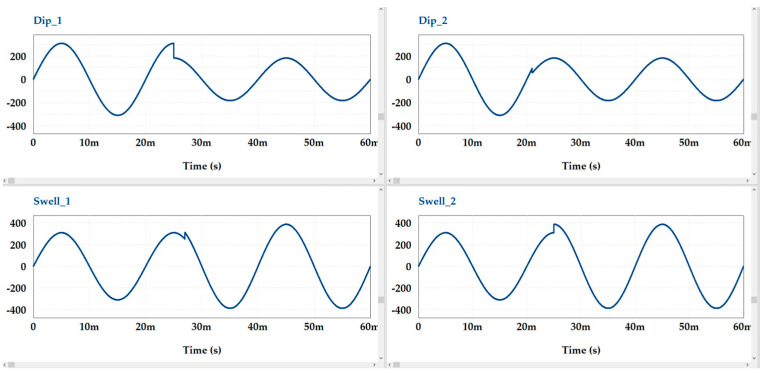
Typical voltage discontinuities are simulated by PSIM software.

The PSIM software can help verify the usefulness of the method. Typical curves of voltage discontinuity inside an AC period are shown in Figure 7. It is worth reminding everyone that the laboratory verification of the method was carried out with a space between 220 and 230 V. In this case, the PSIM simulation matched the laboratory tests. In contrast, the simulated voltage distortions were comparable to those observed in real distribution lines.

PSIM software was successfully used for the simulation of a control system activation for voltage stabilization with a pure sinusoidal signal as well as a signal with voltage discontinuities.

## 3. Results

**Figure 8:**
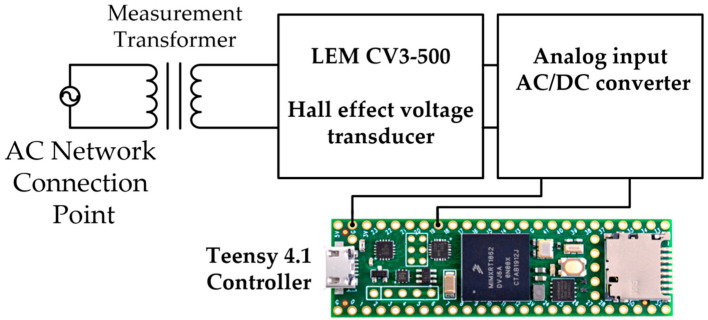
Voltage RMS measurement device with a control system.

**Figure 9:**
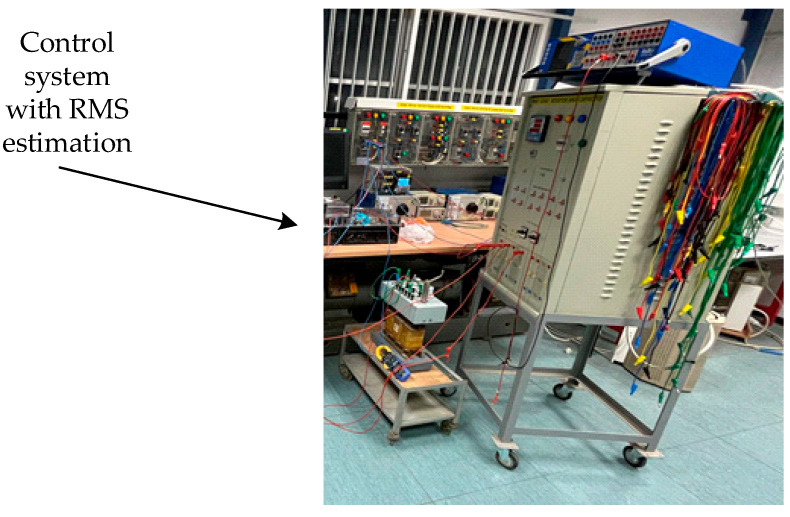
Laboratory stand for the verification of theoretical results.

The results of theoretical RMS predictions were verified in laboratory tests. These tests were carried out on special equipment, including a control system for voltage stabilization (Figure 8), which can quickly recognize voltage RMS, a controllable AC power source, and additional devices simulating the functionality of real high-voltage distribution lines and consumer loads (Figure 9).

The voltage measurement device includes measurement of a high/low voltage transformer, a voltage LEM CV3-500 transducer [29], an analog AC/DC converter, and a microcontroller, Teensy 4.1 [30], responsible for calculation of RMS values during half of the AC period and generating a control signal for voltage correction.

During experiments, the situations that caused voltage discontinuities to occur inside an AC period were simulated by the special controllable power supply DRTS33 [31]. The control system recognized the RMS magnitude of a signal based on the proposed algorithm, and the results of the estimation were compared with the RMS of a sinusoid in the following AC periods. This comparison provided an indicator of the efficacy of the method.

**Figure 10:**
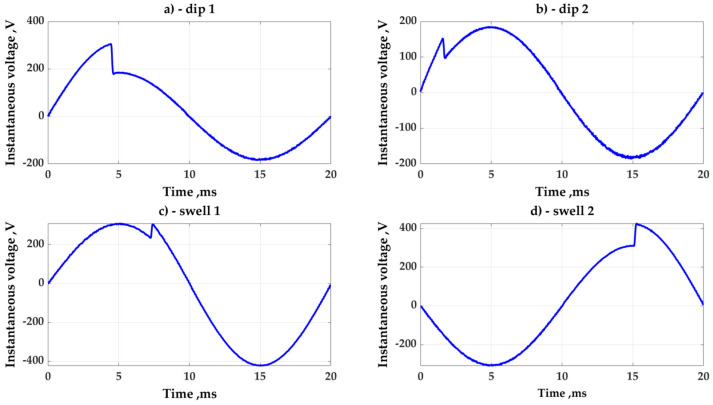
Typical examples for modeling voltage distortion: (**a**,**b**)—voltage dips, (**c**,**d**)—voltage swells.

Typical samples of voltage discontinuities that were simulated are shown in Figure 10a–d.

**Figure 11:**
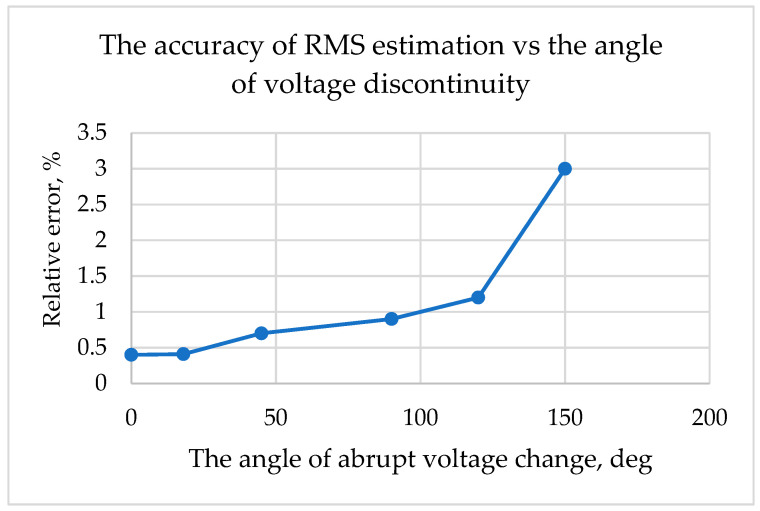
The accuracy of RMS estimation vs. the angle of a voltage distortion.

Results of RMS accuracy vs. the angle *β*_1_ are presented in Table 1 and Figure 11.

**Figure 12:**
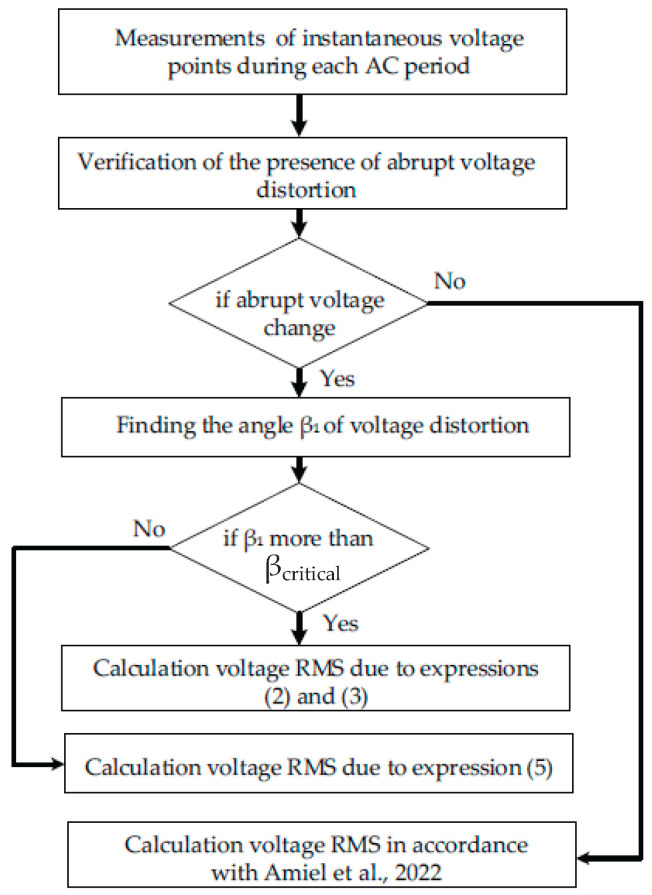
Algorithm of RMS estimation in the presence of voltage distortions [26].

The result of accuracy estimation enabled the development of the algorithm for rapid (during a half of the AC period) RMS assessment in the presence of voltage discontinuities. The algorithm is presented in Figure 12 below.

## 4. Discussion

The rigorous requirements for rapid voltage level stabilization in distribution lines motivated the development of appropriate methods capable of providing an accurate estimation of RMS magnitude during a half-AC period. Existing methods that function satisfactorily for a pure sinusoidal signal give less accurate results for signals containing voltage distortions. The authors modified their original model based on voltage discontinuities. The method proposes two segments for describing a distorted instantaneous voltage signal, each of which acts as a different voltage sinusoid. This approach provides the possibility of defining the RMS of the entire signal during half of the AC period only and developing a strict mathematical expression connecting the RMS value with the magnitudes of the voltages in each segment. The improved RMS estimation approach ensures better accuracy and, as a result, takes less time for the voltage level to stabilize. The estimation error depends on the angle of a voltage discontinuity inside an AC period. When this angle becomes close to ~150° and even greater, the RMS calculation should be modified. The article also suggests methods for RMS assessment in this more extreme situation. However, the accuracy in this case is reduced. When the accuracy of the estimation is insufficient, the correction of the voltage should be delayed until the following half period. Ignoring a correction of low accuracy can cause the loss of control stability and undesirable oscillations of a control signal, which should be avoided. The algorithm for RMS estimation is presented in this work.

The authors intend to continue the improvement of the proposed method to improve its accuracy when the error doesn’t exceed 0.5% for a wider range of angles characterized by the presence of voltage discontinuities.

## 5. Conclusions

As a result of the presented work, a modified method of fast RMS estimation was proposed and demonstrated. The method was developed on the basis of a strict mathematical procedure that included the modeling of a distorted instantaneous voltage, a description of the RMS of the complex signal having some segments with different sinusoidal amplitudes, and a representation of the algorithm ensuring RMS calculation in the presence of voltage discontinuities.

## Figures and Tables

**Table 1:** The error in RMS estimation vs beginning time of abrupt voltage change.

Angle *β*_1_, deg	0	18	45	90	120	150
Rel. error, %	0.4	0.41	0.7	0.9	1.2	3

## Data Availability

Not applicable.
